# Modeling the Impact of Microgravity at the Cellular Level: Implications for Human Disease

**DOI:** 10.3389/fcell.2020.00096

**Published:** 2020-02-21

**Authors:** Peta Bradbury, Hanjie Wu, Jung Un Choi, Alan E. Rowan, Hongyu Zhang, Kate Poole, Jan Lauko, Joshua Chou

**Affiliations:** ^1^Respiratory Technology, Woolcock Institute of Medical Research, Sydney, NSW, Australia; ^2^School of Biomedical Engineering, Faculty of Engineering and Information Technology, University of Technology Sydney, Sydney, NSW, Australia; ^3^Australian Institute for Bioengineering and Nanotechnology, The University of Queensland, Brisbane, QLD, Australia; ^4^State Key Laboratory of Tribology, Department of Mechanical Engineering, Tsinghua University, Beijing, China; ^5^EMBL Australia Node in Single Molecule Science, School of Medical Sciences, University of New South Wales, Sydney, NSW, Australia

**Keywords:** microgravity, mechanobiology, mechanotransduction, cytoskeletal, mechanosensing

## Abstract

A lack of gravity experienced during space flight has been shown to have profound effects on human physiology including muscle atrophy, reductions in bone density and immune function, and endocrine disorders. At present, these physiological changes present major obstacles to long-term space missions. What is not clear is which pathophysiological disruptions reflect changes at the cellular level versus changes that occur due to the impact of weightlessness on the entire body. This review focuses on current research investigating the impact of microgravity at the cellular level including cellular morphology, proliferation, and adhesion. As direct research in space is currently cost prohibitive, we describe here the use of microgravity simulators for studies at the cellular level. Such instruments provide valuable tools for cost-effective research to better discern the impact of weightlessness on cellular function. Despite recent advances in understanding the relationship between extracellular forces and cell behavior, very little is understood about cellular biology and mechanotransduction under microgravity conditions. This review will examine recent insights into the impact of simulated microgravity on cell biology and how this technology may provide new insight into advancing our understanding of mechanically driven biology and disease.

## Introduction

Humans are subjected to persistent gravitational force and the importance of gravity for maintaining physiological function has been revealed by the detrimental impacts of space travel on human health. During space flight, astronauts are exposed to a prolonged state of microgravity and develop a myriad of physiological disruptions including a loss of muscle mass and bone density, impaired vision, decreased kidney function, diminished neurological responses, and a compromised immune system (White and Averner, 2001; Horneck et al., 2003; Crucian et al., 2014; White et al., 2016). This review will discuss recent data that highlight the impact of microgravity at the cellular level. Additionally, this review addresses how such research can be conducted on earth by simulating the microgravity state. These studies are not only important for understanding how humans are affected by microgravity but have the potential to elucidate the role of mechanical stimuli on cellular function and the development of mechanically driven disease states.

Mechanobiology is the study of how cells are influenced by their physical environment. This emerging field of research provides an important perspective on understanding many aspects of cellular function and dysfunction. Cells can convert mechanical inputs into biochemical signals to initiate downstream signaling cascades in process known as mechanotransduction. Gravitational force is presumed to play a crucial role in regulating cell and tissue homeostasis by inducing mechanical stresses experienced at the cellular level. Thus, the concept of mechanical unloading (a decrease in mechanical stress) is associated with the weightlessness of space and can be replicated by simulating microgravity conditions, allowing for investigation of the mechanobiology aspects of cell function. The mechanical unloading of cells under microgravity conditions shifts the balance between physiology and pathophysiology, accelerating the progression and development of some disease states. For example, kidney stone formation is accelerated under microgravity conditions compared to Earth’s gravity (1 g) (Pavlakou et al., 2018). Similarly, osteoporosis can take decades to develop under normal gravitational loading, yet this disease can be modeled under microgravity over shorter time scales (Pajevic et al., 2013). The mechanisms by which human physiology are disrupted in microgravity remain unknown, rendering numerous open questions regarding the adaptive changes that occur at the cellular and molecular level in response to microgravity.

## Simulating Microgravity

One of the key challenges in using microgravity as an investigative tool is creating a microgravity condition that can be applied at a cellular level, on Earth. The process of conducting space research missions is costly and time consuming, thereby limiting the advancement of microgravity research and widespread application of this approach. Currently, there are a number of microgravity devices available for purchase that are designed to achieve microgravity conditions. Microgravity simulators specific to cellular studies include strong magnetic field-induced levitation (i.e., diamagnetic simulation), as well as two-dimensional and three-dimensional clinostats, rotating wall vessels and random positioning machines (RPMs) (Huijser, 2000; Russomano et al., 2005; Herranz et al., 2012; Ikeda et al., 2017). Each of the simulation techniques has shown advantages and disadvantages however, when chosen correctly for a given experiment, the results obtained are similar to those observed in Space flight studies (Stamenkovic et al., 2010; Herranz et al., 2013; Martinez et al., 2015; Krüger et al., 2019b). For cell culture studies, the use of RPM is common as the system achieves microgravity by continually providing random changes in orientation relative to the gravity vector and thus an averaging of the impact of the gravity vector to zero occurs over time (Beysens and van Loon, 2015). This averaging is achieved by the independent, yet simultaneous, rotation of two axes – with one axis rotating in the *X*-plane, and the second axis rotating in the *Y*-plane. It is important that the cell culture flask/sample be placed at the midpoint of the *x*-axis, cell culture flasks placed at a distance from the center of the *x*-axis will be subjected to a greater rotational force resulting in cells experiencing both centrifugal force and an increased gravity load (Beysens and van Loon, 2015). Furthermore, the RPM is designed to subject the cells to 10^–3^ g (or as close to this value as possible) but cannot achieve complete zero gravity and hence termed microgravity (Huijser, 2000; Beysens and van Loon, 2015).

## The Impact of Microgravity of Cell Cytoskeleton

Cellular response to mechanical loading has been well documented over the decades however, the response that occurs when cells are placed under conditions of mechanical unloading remains in its infancy. The most apparent cellular changes that occur following exposure to a microgravity environment are alterations to cell shape, size, volume, and adherence properties (Buken et al., 2019; Dietz et al., 2019; Thiel et al., 2019b). These microgravity induced changes to cellular morphology reflect modifications to cytoskeletal structures, namely microtubules and actin filaments (F-actin), as cells sense a reduced gravitational load and therefore mechanical unloading (Crawford-Young, 2006; Corydon et al., 2016a; Thiel et al., 2019a). Microgravity, whether in Space or simulated in the laboratory, offers a unique mechanical unloading environment to explore cellular mechanotransduction by providing an unparalleled research environment to investigate the relationship between mechanical unloading and cellular response.

Numerous studies have been conducted on a myriad of cell types highlighting morphological sensitivity to microgravity (Ingber, 1999; Vorselen et al., 2014), with the first documented morphological change reported by Rijken et al. (1991). Morphological changes as a result of microgravity conditions, either real or simulated, have been shown to have altered transcription, translation, and organization of cytoskeletal proteins (Vassy et al., 2001; Infanger et al., 2006b; Tauber et al., 2017). Fundamental work carried out by Tabony, Pochon, and Papaseit showed that while tubulin self-assembly into microtubules occurs independent of gravity, the assembly and organization of the microtubule network is gravity dependent (Papaseit et al., 2000; Tabony et al., 2002). Importantly, this gravity-dependent organization of the microtubule network has since been described in multiple cell lines during both real and simulated microgravity exposures (Vassy et al., 2001; Uva et al., 2002; Hughes-Fulford, 2003; Rosner et al., 2006; Janmaleki et al., 2016) and possibly be the result of a poorly defined microtubule organizing center (MTOC) (Lewis et al., 1998). Taken together these data highlight an important regulatory role for the microtubule network and the MTOC following exposure to a microgravity environment. However, the data surrounding the response of the actin cytoskeleton to microgravity exposure are less clear. Many studies have reported that microgravity exposure had decreased expression of actin and actin-associated proteins, namely Arp2/3 and RhoA, subsequently resulting in the disorganization of the actin cytoskeleton (Carlsson et al., 2003; Higashibata et al., 2006; Corydon et al., 2016a, b; Louis et al., 2017; Tan et al., 2018). However, other studies have showed increased F-actin and stress fiber formation that accompanied the development of lamellipodia protrusions following exposure to microgravity (Gruener et al., 1994; Nassef et al., 2019). Contrary to this, Rosner et al. (2006) reported no changes to actin structure or organization and further suggested that the actin cytoskeleton is only regulated in a hyper-gravity environment. Thus, the data surrounding cellular morphological changes in response to microgravity and the role of actin is confounding and at times contradictory.

The actin cytoskeleton, its organization and ability to generate force are critical for cellular mechanosensing and importantly any changes to these processes can initiate pathophysiological disruption. Transduction of mechanical forces by integrins requires clustering of these transmembrane receptors and the subsequent formation of focal contacts and adhesions that physically link the extracellular matrix (ECM) to the cytoskeleton (Wang et al., 1993; Ingber, 1997; Maniotis et al., 1997). Binding of integrins to matrix proteins promotes the bundling of F-actin at the cell-matrix adhesion and the subsequent maturation of both the focal adhesion and the actin stress fiber (Zaidel-Bar et al., 2003; Wolfenson et al., 2009, 2011) to generate the tension required for cell adherence, migration, and tissue homeostasis. Exposure to microgravity reduces the formation, number, and total area of focal adhesions per cell (Guignandon et al., 2003; Tan et al., 2018) consequently affecting cellular adherence, migration capacity, and viability (Plett et al., 2004; Nabavi et al., 2011; Shi et al., 2015; Ahn et al., 2019; Dietz et al., 2019), albeit with contradictory results. Mechanical unloading has been shown to significantly reduce gene expression of a number of focal adhesion proteins, including FAK, DOCK1, and PTEN, while caveolin and p130Cas expression were shown to be increased (Grenon et al., 2013; Ratushnyy and Buravkova, 2017). Thus, the activity of the downstream signaling pathways that govern the microgravity-induced cytoskeletal changes are significantly impaired and are at least in part due to the microgravity-triggered inhibition of FAK and/or RhoA signaling (Higashibata et al., 2006; Li et al., 2009; Tan et al., 2018). Furthermore, recent data suggest that changes to the cytoskeleton may also impact signaling via mechanically activated ion channels and contacts in response to both cell-generated (Nourse and Pathak, 2017; Ellefsen et al., 2019) and externally applied mechanical inputs (Bavi et al., 2019). Thus, downstream signaling of the numerous mechanotransduction pathways depend on the concerted interaction of the cytoskeleton, cell adhesion molecules, and force sensing proteins, including mechanically activated ion channels. To date, there is little information regarding the role of mechanically activated ion channels in microgravity environments.

## Microgravity Impact Bone Cell Signaling Response and Cartilage ECM Synthesis

The accelerated loss of bone and muscle mass as a result of microgravity has been well documented over the decades (Burger and Klein-Nulend, 1998; Harris et al., 2000; Fitts et al., 2001). Osteocytes and osteoblasts are known mechanosensitive bone cells responsible for maintaining the balance of bone absorption and resorption – a process that is coordinated by both the actin cytoskeleton and microtubule network (Okumura et al., 2006). Bone cell morphology is significantly modified following exposure to microgravity when compared to control cells (Guignandon et al., 1995; Hughes-Fulford, 2003). To adapt to the new mechanical environment the bone cells have reduced transcription and translation of cytoskeletal and cytoskeletal-associated proteins (Xu et al., 2017; Mann et al., 2019), decreased focal adhesion formation, together resulting in the increased formation of osteoclast resorption pits (Nabavi et al., 2011). Furthermore, the actin cytoskeleton of osteoblasts subjected to 4 days of microgravity exposure completely collapsed (Hughes-Fulford, 2003), significantly impacting multiple downstream signaling pathways, most notably, the inhibition of bone morphogenic protein (BMP) signaling axis (Patel et al., 2007; Xu et al., 2017). The BMP family of proteins regulates the expression of an important mechanosensing protein, sclerostin, found exclusively in osteocytes (Poole et al., 2005; Kamiya et al., 2016), whereby mechanical unloading increases sclerostin protein expression to promote bone resorption and cause a loss of bone density (Robling et al., 2008) – a phenotype that closely mimics both osteoporosis and osteonecrosis. Thus by applying the unique mechanical unloading environment offered by both real and simulated-microgravity to bone (specifically, osteoporosis) research has led to the introduction of the FDA approved drug, Evenity, a monoclonal antibody that works as an anabolic agent to increase bone mass via the sclerostin pathway (Scheiber et al., 2019).

While a significant number of studies have identified the importance of mechanical unloading in regulating bone structure and function, the articular cartilage (AC) is also particularly susceptible to the effects of mechanical loading and unloading (Sanchez-Adams et al., 2014). The cells found in AC, chondrocytes, sense and respond to changing mechanical loads in order to maintain the balanced production of ECM molecules ensuring that the tissue maintains the ability to resist tensile and compressive forces. Both mechanical unloading and overloading of chondrocytes can disrupt the homeostatic balance in the cartilage leading to cartilage degradation and osteoarthritis thereby tipping the balance from homeostatic maintenance to pathophysiology, leading to cartilage degradation and osteoarthritis (Vanwanseele et al., 2002; Kurz et al., 2005). To study the effect of extended microgravity on AC specifically on the joint tissue, mice were exposed to 30 days of spaceflight during the Bion-M1 mission (Fitzgerald et al., 2019). Interestingly, tissue degradation was observed only in the AC of load-bearing joints, but not in minimally loaded sternal fibrocartilage highlighting a differential response to mechanical unloading and the predisposition of load bearing joints, but not structural joints, to mechanical stimuli. Additionally, decreased proteoglycan levels were found in the AC of the mice after the 30 days (Vanwanseele et al., 2002) further characterizing a mechanical unloading pathology specific to AC atrophy. Importantly, reduced proteoglycan levels have also been reported in hindlimb unloading and limb immobilization studies in various animals (Salter et al., 1980; Haapala et al., 1996; O’Connor, 1997). The reduced proteoglycan levels paired with the augmented regulation of ECM-associated genes and proteins that help protect against osteoarthritic changes, including collagen type I, II, and X, β_1_integrin, vimentin, and chondrocyte sulfate (Ulbrich et al., 2010; Aleshcheva et al., 2013, 2015), suggest that while the microgravity-induced osteoarthritic pathology is observed cartilage recovery of the AC is possible. Further to this, cell-based studies have shown that primary chondrocytes are to adapt to a microgravity environment within 24 h (Aleshcheva et al., 2013). There is a clear need for more research into the response of AC and specifically chondrocytes as elucidation of the molecular mechanism that underpins chondrocytes mechanoadaptation to a microgravity environment, holds great promise for novel osteoarthritic treatments.

## Microgravity-Induced Cytoskeletal Regulation of Immune and Cancer Cells

The function of the immune system is strongly impacted (Frippiat et al., 2016; Smith, 2018) with several studies reporting dysregulation or immunosuppression following simulated or real microgravity conditions (Boonyaratanakornkit et al., 2005; Crucian et al., 2015; Martinez et al., 2015; Thiel et al., 2017). Peripheral monocytes collected from astronauts post short-duration Space missions (13–16 days) showed that there was no change in the numbers of circulating monocytes indicating that the change to an immunosuppressive phenotype was not due a reduced cell number (Crucian et al., 2011). However, peripheral monocytes showed a significantly decreased expression of surface markers CD26L and HLA-DR, known regulators of lymphocyte-endothelial cell adhesion and tissue migration (Crucian et al., 2011). *In vitro* studies performed during Space flights have revealed that lymphocytes exhibit important changes in their cytoskeletal properties, suggesting that T cell activation may be compromised at the level of the T cell receptor (TCR) interaction (Sonnenfeld et al., 1992). It has been hypothesized that immunosuppression produced in microgravity is due to impaired TCR activation resulting from cytoskeletal disruption (Bradley et al., 2019); however, the underlying molecular mechanisms remain unknown.

**FIGURE 1:**
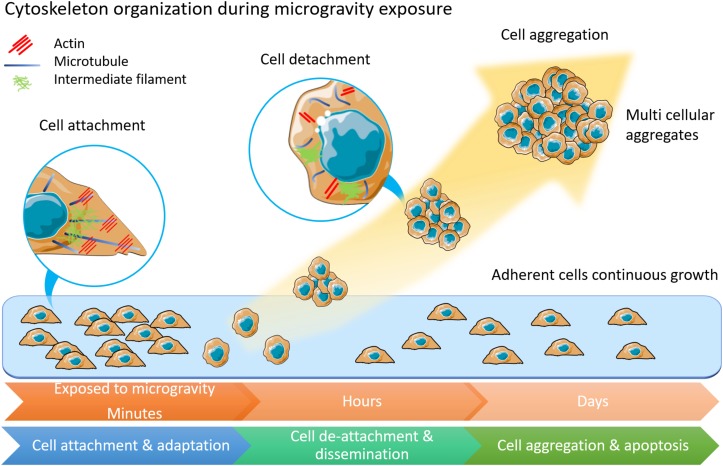
The morphology and physiology alterations of adherently growing cells after microgravity exposure. Cytoskeleton components of actin, microtubules and intermediate filament are displayed in inset circles. In adherent cells, microtubules form radiation arrangement near nuclear. Actin fibers anchor to cell membranes. Intermediate filament forms loose network around nuclear. Among cells under microgravity influence, the microtubules are shortened and curved. Less actin fibers but more condense intermediate filament are observed. This illustration was inspired by long-term thyroid cells culture in simulated microgravity environment (Kopp et al., 2015; Krüger et al., 2019a).

When applied to tumor cells microgravity has been found to impact tumor cell adhesion, proliferation, migration, and viability (Grimm et al., 2002; Plett et al., 2004; Infanger et al., 2006a; Shi et al., 2015; Tan et al., 2018), and to induce cell autophagy (Jeong et al., 2018). Changes in apoptotic rate were also observed in colorectal cancer cells (DLD-1) and a lymphoblast leukemic cell line (MOLT-4), accompanied by reduced transcription of the genes involved in colony formation, oncogenic progression, and metastatic potential (Vidyasekar et al., 2015). The foremost changes to tumor cell following exposure to microgravity are alterations of cell shape, size, and adhesion, indicating changes in cytoskeletal organization (Figure 1). Modulation of the cytoskeletal network have been demonstrated to occur after just minutes (Rijken et al., 1992; Sciola et al., 1999) or hours (Lewis et al., 1998; Vassy et al., 2001) in microgravity. Microtubule disorganization was observed in both the breast cancer MCF-7 cells and the thyroid cancer cell line FTC-133 when exposed to real microgravity (Kopp et al., 2018a, b). In contrast, no changes in Rac-controlled F-actin were detected in the neuroblastoma cell line, SH-Y-5Y (Rosner et al., 2006), highlighting the differing responses of distinct cell types to mechanical unloading. The expression of focal adhesion proteins, moesin and ezrin, was found to be significantly down-regulated after 24-h of microgravity exposure (Kopp et al., 2018a). There remains a gap in the understanding of the molecular mechanisms that drive changes in the cytoskeleton in response to mechanical unloading and the physiological systems that will be impacted by microgravity. Thus, there is a dual potential of microgravity studies in both elucidating the underlying importance of mechanical signaling in human physiology and in developing understanding and countermeasures for long-duration space flights.

## Discussion

Microgravity research conducted on the International Space Station (ISS) and in simulated microgravity has highlighted the importance of cellular mechanotransduction in human health and disease (Table 1). Understanding the molecular mechanisms by which cells respond to mechanical unloading will not only be important for preparing humans for longer-term space exploration but may also contribute to therapeutics for the treatment of diseases that depend on mechanical interactions, highlighting opportunities to manipulate and correct certain disease states. The discovery of sclerostin and the subsequent generation and use of the sclerostin monoclonal antibody to treat both osteoporotic patients has been a major outcome of the field (Martin et al., 2020). Thus, Space and microgravity biological research constitute extreme environments in which novel mechanotransduction molecules and mechanosensing mechanisms can be identified and may prove helpful in better designing immunotherapies or developing better and more targeted anti-cancer therapies. Studies that leverage these low gravity environments have the unique potential to unveil important physiological changes that occur in response to changing mechanical loads and are of considerable importance in expanding our understanding of mechanobiology.

**TABLE 1:** Effects of sub-cellular functions concerning various cell types and exposure duration in microgravity environment.

**Cell type**	**Effects of cells**	**Microgravity exposure time**	**References**
Osteosarcoma cells (ROS 17/2.8)	Cell morphological change to rounded shape with long cytoplasmic extensions	4 days and 6 days	Guignandon et al., 1997
Osteosarcoma cells (ROS 17/2.8)	Reduction in cell spread area and vinculin spot area, actin and focal adhesion, and stress fibers	12 and 24 h	Guignandon et al., 2003
Breast cancer (MCF-7)	Disoriented microtubule	1.5 h	Vassy et al., 2003
Thyroid cancer (ML-1)	Actin fiber reorganization	Parabola flight	Ulbrich et al., 2011
Human macrophages	No effect on cytoskeletal structure	11 days	Tauber et al., 2017
Human chondrocytes	Effect on cell cytoplasm, microtubule network disruption, loss of stress fibers, actin fiber reorganization.	Parabola flight	Aleshcheva et al., 2015
Osteoblasts (MC3T3-E1)	Reduction in actin cytoskeletal stress fibers and reduction of nuclei size by 30%	4 days	Hughes-Fulford and Lewis, 1996
Primary mouse osteoblasts	Thicker microtubule, smaller focal adhesion spots, reduction in actin stress fibers, and increase in cell spread area	5 days	Nabavi et al., 2011
Osteocytes	Increase in cellular organelles including Golgi complex, vacuoles, and vesicles	14 days	Rodionova et al., 2002

With the privatization and commercialization of the ISS and a global push toward the exploration of space, the gateway for conducting research under simulated and Space microgravity is becoming more accessible. In particular the development of different variations of the RPM device provides a simulated microgravity environment on Earth for investigating the changes in cellular function due to mechanical unloading. Over the last several years, experiments involving the use of microgravity to study cellular mechanobiology and disease mechanisms have continued to rise, reinforcing the importance of this platform. This area of research has highlighted the importance of mechanical cues in maintaining cell and tissue homeostasis. The emergence of Space mechanobiology will continue to rise in the foreseeable future as it is evident that the benefits of such research can catapult survival of astronauts in space for extended duration as well as developing understanding and better treatments for Earth-borne diseases.

## Author Contributions

PB contributed to the sections “Simulating Microgravity” and “The Impact of Microgravity of Cell Cytoskeleton.” JuC and JL contributed to the section “Microgravity Impact Bone Cell Signaling Response and Cartilage ECM Synthesis.” HW, HZ, and KP contributed to the section “Microgravity-Induced Cytoskeletal Regulation of Immune and Cancer Cells.” AR, KP, and JoC initiated, conceptualized the review, and edited the manuscript.

## Conflict of Interest

The authors declare that the research was conducted in the absence of any commercial or financial relationships that could be construed as a potential conflict of interest.
